# Medical Portraiture

**Published:** 1911-01-28

**Authors:** C. Gerald Harmer

**Affiliations:** College of Physicians and Surgeons, Baltimore


*To the Editor of* The Hospital.

### MEDICAL PORTRAITURE.

To the Editor of The Hospital.

Dear Sir,—It was a pleasure to read your notice relative to "Medical Portraiture" on p. 337 of the issue of December 17, 1910; especially interesting was it on account of the fact that for some time past it has been my endeavour to obtain for the library of this college portraits of the great men in medicine. I believe that considerable stimulus to a student's aspiration may be induced by his becoming familiar with the biography and portrait of the great teachers or practitioner of medicine.

As an Englishman, it is with regret that I find that many of the great men in the profession in England are not any way near as well known as are many on the Continent, and it is for that reason that I ask you to give to me as complete information as you may have as to where I might be able to obtain copies of the portraits of any and all the great minds in medicine in Great Britain—those passed away and those living. I am anxious to get as many as possible, which I intend (after obtained) to be presented to the college and to be hung in the library. If you can also furnish me the names of the publishers of the biographies of the great men in medicine I should be thankful. If I could get them I would present these to the library. Any assistance that you may be able to render me in my endeavour will be most gratefully appreciated.

My thanks to you in advance; appreciating hearing from you.—I am, very truly yours,

College of Physicians and Surgeons, Baltimore, January 11. C. Gerald Harmer.

[We hope to publish a series of articles on the historical portraits of English medical men shortly; meanwhile we shall be grateful to correspondents who can give us information with regard to existing portraits not only to enable us to answer Dr. Harmer's inquiry, but to aid us in dealing properly with the whole subject.—Ed. The Hospital.]

